# Microstructure and Thermal Reliability of Microcapsules Containing Phase Change Material with Self-Assembled Graphene/Organic Nano-Hybrid Shells

**DOI:** 10.3390/nano8060364

**Published:** 2018-05-24

**Authors:** Xianfeng Wang, Yandong Guo, Junfeng Su, Xiaolong Zhang, Ningxu Han, Xinyu Wang

**Affiliations:** 1 Guangdong Provincial Key Laboratory of Durability for Marine Civil Engineering, Shenzhen University, Shenzhen 518060, China; xfw@szu.edu.cn; 2 Department of Polymer Material, School of Material Science and Engineering, Tianjin Polytechnic University, Tianjin 300387, China; ydguo008@163.com (Y.G.); xlzhang008@163.com (X.Z.); 3 School of Mechanical Engineering, Tianjin University of Commerce, Tianjin 300134, China; wxy@tjcu.edu.cn

**Keywords:** Nano-hybrid, microcapsule, phase change material, graphene, thermal conductivity

## Abstract

In recent decades, microcapsules containing phase change materials (microPCMs) have been the center of much attention in the field of latent thermal energy storage. The aim of this work was to prepare and investigate the microstructure and thermal conductivity of microPCMs containing self-assembled graphene/organic hybrid shells. Paraffin was used as a phase change material, which was successfully microencapsulated by graphene and polymer forming hybrid composite shells. The physicochemical characters of microPCM samples were investigated including mean size, shell thickness, and chemical structure. Scanning electron microscope (SEM) results showed that the microPCMs were spherical particles and graphene enhanced the degree of smoothness of the shell surface. The existence of graphene in the shells was proved by using the methods of X-ray photoelectron spectroscopy (XPS), transmission electron microscopy (TEM), and atomic force microscopy (AFM). It was found that graphene hybrid shells were constructed by forces of electric charge absorption and long-molecular entanglement. MicroPCMs with graphene had a higher degradation temperature of 300 °C. Graphene greatly enhanced the thermal stability of microPCMs. The thermal conductivity tests indicated that the phase change temperature of microPCMs was regulated by the graphene additive because of enhancement of the thermal barrier of the hybrid shells. Differential scanning calorimetry (DSC) tests proved that the latent thermal energy capability of microPCMs had been improved with a higher heat conduction rate. In addition, infrared thermograph observations implied that the microPCMs had a sensitivity response to heat during the phase change cycling process because of the excellent thermal conductivity of graphene.

## 1. Introduction

The whole world is currently confronted with a global energy crisis in that the increasing world population is linked with a large increasing energy demand. One of the effective ways to reduce energy demands is the use of thermal energy storage [1]. Depending on environmental circumstances, phase change materials (PCMs) can absorb, store, and release large latent heat decreasing the gap between energy supply and energy consumption [2]. Over the last decades, latent heat energy storage has been an attractive method and has been paid much attention for heating and cooling purposes in many fields, such as solar thermal conversion, building energy saving, and thermal energy storage [3]. Many organic and inorganic PCMs have been applied in the field of latent heat storage including polyols, fatty acids, alkane, and hydrated inorganic salts [4]. Organic PCMs, especially long-chain alkanes, have lots of advantages such as being innocuous while having low supercooling and high latent heat. These long-chain alkane PCMs (octadecane, eicosane, docosane, octacosane) do keep their shape easily because they are liquids at high temperature. To overcome this disadvantage, microencapsulation of PCMs (microPCMs) is an alternative approach [5]. MicroPCMs can prevent leakage of the melted PCMs during a phase change process, reduce the PCMs reactivity with the outside environment, enlarge the heat transfer area, and increase the heat transfer rate [6]. MicroPCMs are defined as particles owing a core-shell structure with a diameter less than 1000 μm. Normally, the shell is polymer material fabricated using polymerization through methods of in-situ polycondensation, suspension polycondensation complex coacervation, and interfacial polycondensation [7]. The widely used polymers are melamine-formaldehyde resin, polyurea-formaldehyde resin, polystyrene and poly(methylmethacrylate) because they are cheap and easily form shells [7].

A literature review shows that microPCMs have been widely studied including the fabrication process, microstructure control, mechanical properties, and latent heat storage properties [8]. Less attention has been paid to improve the microPCMs with regard to a higher thermal conductivity and higher thermal stability, especially for the microPCMs with polymeric shell. For the core materials such as alkane materials, their thermal conductivity is only about 0.2–0.3 W/(m∙K), which may reduce the rate of heat storage and extraction during the melting-solidification cycles [9]. On the other hand, the polymeric shells resist heat transition at the same time. A challenging problem which arises in this domain is to enhance the thermal conductivity of the shells. Therefore, heat transfer enhancement is of theoretical importance as well as for practical significance toward the performance promotion of thermal energy storage systems by increasing the effective thermal conductivity of PCMs [10]. The improvement of microPCMs thermal properties and especially the effective thermal conductivity by microstructure design is therefore of particular interest [11]. One approach is to improve the thermal conductivity of the core materials. Testing results have proved that unidirectional solidification of composite PCMs is accelerated in proportion to the increased thermal conductivity [12]. This means that the homogenous assumption of effective thermo-physical properties may be valid for modeling the heat transfer of composite PCMs disregarding the instability issue in practice. The dynamics of nanoparticles during solidification and their interactions with the moving solid-liquid interface have been studied by considering the transport of nanoparticles by an extended Stefan problem formulation. Based on the above-mentioned results, metal oxide nanoparticles and carbon nanotubes, graphite and graphene have been employed as the nano-fillers applied in the melting of composite PCMs [13]. It has been found that carbon-based nano-materials are sound filler materials due to their inherently high thermal conductivity and low density. The filler-induced alignment of the PCM molecules has been identified as contributing to the pronounced thermal conductivity increase of carbon nano-fillers.

Another approach is to enhance the thermal conductivity of polymeric shell materials through nano-filler additives [14]. More precisely, the influence of the heating rate taking into account the investigated shell structure has been reported in very few papers and more research is particularly needed. Thus, in order to control the heat transmission performance of the PCM-based material, the inherent properties of the shells should be well known with sufficient accuracy. Without any doubt, the enhancement of thermal conductivity of microPCMs via additives in the polymeric shells is one of the most important modern developments in engineering technology aiming at increasing the coefficient of heat transfer [13]. Metal particles and inorganic particles have been applied to form a composite shell structure to enhance thermal conductivity of microPCMs [15]. The thermal conductivity of solid metals was higher than polymer material, so the suspended metal particles were able to increase the thermal conductivity and heat transfer performance. Su et al. [16] also proved that the addition of nano-CaCO_3_ enhanced the thermal conductivity of microPCMs. In spite of such promising characteristics, there exists rare reportage in the literature addressing the use of this method for improving thermal conductivity.

Graphene, as a 2D carbon material, has a superior thermal conductivity of 5000 W/(m∙K) and a high specific surface area of 2630 m^2^/g [17]. It is a promising element to improve the thermal conductivity of PCM, and some work in this field has been done. For example, a phase change material composed of three-dimensional graphene aerogel and octadecanoic acid was fabricated and the tests showed that the thermal conductivity of the composite increased about fourteen times but the latent heat of the composite had a 4.3 J/g decrease compared to pure PCM. Li et al. [18] prepared a docosane-based phase change material in the presence of spongy graphene. The latent heat and thermal conductivity of docosane/graphene composites were simultaneously enhanced in contrast to that of pure docosane. The latent heat increased from 256.1 J/g to 262.8 J/g, and the thermal conductivity increased more than twice as much with a low graphene concentration of 3.0 mg/cm^3^. It was reported that graphene nano-platelets were dispersed in liquid eicosane at various loadings of 0–10 wt %. The thermal conductivity enhancement was above 400% for the highest loading of 10 wt %, whereas the latent heat of the PCM composite correspondingly decreased by 16% [18]. As shown in the above results, the latent heat of the PCM composites generally decreased with the addition of graphene, though their thermal conductivity increased. Zhang et al. [19] fabricated microPCMs with a graphene shell. It was found that the microPCMs had a relatively spherical shape with 0.05–0.10 wt % graphene. The heat transfer performance, thermal stabilities, and anti-osmosis performance of the microPCMs showed a remarkable improvement. It needs to be mentioned that grafted graphene oxide also has been successfully applied to enhance the thermal properties of microPCMs [20].

Nowadays, graphene nano-sheets are famous for their outstanding properties such as high thermal conductivity and ultra-low electrical resistivity. The most popular route for the bulk production of graphene sheet is the chemical oxidation and exfoliation of graphite, followed by reduction. Interestingly, the low density of graphene sheets is another advantage for making it a good choice to modify the microcapsules [18,19,20]. However, few works have been carried out to investigate the microstructure of polymer/graphene shells. Moreover, it is necessary to understand the effects of graphene on the thermal conductivity of microPCMs. Heat transmission details through the polymer/graphene shells should be given to make the thermal physiochemical mechanism clear. The method of graphene addition in shells plays a vital role in the physical properties of microPCMs especially the thermal conductivity. Based on the above analysis, the aim of this study was to investigate the microstructure and thermal conductivity of microPCMs containing paraffin with graphene/organic hybrid shells. The effect of graphene content in the shells on the thermal transmission was evaluated by a thermal absorbing-releasing cycle method. At the same time, the relationship was also discussed between the microstructure changes and the thermal performances of the microPCMs.

## 2. Experimental

### 2.1. Materials

Paraffin was used as the PCM material (0.889 g/cm^3^, Tianjin Kemel Chem. Co., Ltd., Tianjin, China). Methanol modified melamine-formaldehyde (MMF) prepolymer (Aonisite Chemical Trade Co., Ltd., Tianjin, China) was used as the polymer shell material with solid content of 78.0%. Styrene maleic anhydride (SMA) copolymer (Scripset©520, Hercules, CA, USA) was applied as a dispersant. Graphene was supplied by Huifeng Co., Ltd. (Changsha, Hunan Province, China, ≥85.0 wt %). All other chemicals, unless otherwise stated, were obtained from commercial sources and used as received.

### 2.2. Synthesis of microPCMs with Graphene

The microPCMs were prepared by a self-assembled graphene/organic hybrid polymerization method. In details, the whole process can be divided into three steps: (a) SMA (2 g) was added to 100 mL water at 50 °C and allowed to mix for 2 h. Then a solution of NaOH (10 wt %) was added dropwise adjusting its pH value to 10. The above surfactant solution and paraffin (50 g) were emulsified mechanically with a vigorous stirring rate for 10 min using a high-speed disperse machine. (b) The encapsulation was carried out in a 500 mL three-neck round-bottomed flask equipped with a condenser and a tetrafluoroethylene mechanical stirrer. The above emulsion was transferred to the bottle, which was dipped in a steady temperature flume (room temperature). The mixture of MMF prepolymer (25 g) and various contents of graphene were added dropwise with a stirring speed of 500 r·min^−1^. The temperature was increased to 80 °C with a rate of 2 °C·min^−1^. (c) After polymerization for 1 h, the temperature was decreased slowly to ambient temperature. Finally, the resultant microcapsules were filtered and washed with pure water and dried in a vacuum oven.

### 2.3. Morphology and Geometry of microPCMs

Optical photographs of the microPCMs retained in the emulsion were taken by an optical microscope (MiVnt image analyze system, Shanghai, China). The surface morphologies of dried microPCMs were observed by using a Scanning Electron Microscope (SEM, FEI Nanosem 430, Hillsboro, OR, USA) at an accelerated voltage of 15 kV. The mean size of microPCMs was measured using a partial diameter distribution machine (LA950 V2, Horiba, Tokyo, Japan). The measurement method of shell thickness was applied in previous work [14]. About 10 g MMF-shell microPCMs was mixed in 50 g gelatin solution (30 wt %, Tianjin Sinogo Co., Tianjin, China). After the composite was dried in room temperature, it was carefully cut to obtain the cross-section by an ultramicrotome (RMCMT-7000, Boeckler Instruments Inc., Tucson, AZ, USA). The shell thickness was an average value of 50-times measurement results for each microcapsule sample.

### 2.4. Microstructure Analysis of Shells

Fourier-Transform infrared (FTIR) spectra of dried films were obtained using a Nicolet Magna 750 spectrometer with a deuterated triglycine sulfate detector and Omnic 3.2 s software. Scanning was carried out in the range 4000–400 cm^−1^ with a resolution of 4 cm^−1^, and 128 scans were averaged for each sample.

A transmission electron microscope (TEM, Hitachi HT7700, Japan) was utilized to observe the graphene on the surface of microPCMs. Atomic force microscopy (AFM) examination was carried out for each microcapsule sample in the tapping mode with a NanoScope V AFM (Digital Instruments, Veeco) [14]. The elemental ratio of microPCMs was characterized by X-ray photoelectron spectroscopy (XPS, ESCALAB™ 250Xi, ThermoFisher, Waltham, MA, USA).

### 2.5. Thermal Stability of microPCMs

The thermal stability characterization of microPCMs was performed on a Dupont SDT-2960 thermogravimetric analysis (TGA) at a scanning rate of 5 °C·min^−1^ in a flow of 40 mL·min^−1^ nitrogen (N_2_). Another method was adopted to evaluate the thermal stability of microPCMs by a thermal absorbing-releasing process using a temperature-controlled chest [21]. The samples were heated to 50 °C with a rate of 2 °C·min^−1^ and kept for 10 min, and then the temperature decreased to −10 °C·min^−1^ with the same rate of 2 °C·min^−1^. This heat absorbing-releasing process for each sample was repeated for 50, 100, 150, and 200 cycles. The final states of microPCMs were observed by SEM to analyze their thermal stability.

### 2.6. Thermal Conductivity of Microcapsules

The thermal conductivity values of microPCMs samples were measured using a thermal conductivity instrument (DRXL, Shanghai Jiezhun Instrument Equipment Co., Ltd. Shanghai, China)*.* The tests were in accordance with ASTM E-1530 standard. A powder sample (20 g) was held under a uniform compressive load between two polished surfaces, each controlled at a different temperature. The lower surface was part of a calibrated heat flow transducer. The heat flowed from the upper surface, through the sample, to the lower surface, establishing an axial temperature gradient in the stack. After reaching thermal equilibrium, the temperature difference across the sample was measured along with the output from the heat flow transducer. These values and the sample thickness were then used to calculate the thermal conductivity. The temperature drop through the sample was measured with temperature sensors in the highly conductive metal surface layers on either side of the sample. For one-dimensional thermal conduction the formula can be given as Equation (1),

(1) $$Q=\lambda A\frac{T_{1}-T_{2}}{x}$$

where *Q* is the heat flux (W), λ is the thermal conductivity (W/(m·K)), *A* is the cross section area (m^2^), *x* is the thickness of the composite sample (m). The *Q* value is the average of three tests under the same conditions.

### 2.7. Infrared Thermograph Observation

An infrared thermograph (IRT, HT-02, XinTest, Shenzhen, China) was applied to measure the thermal transmission of various microPCMs with graphene. Each microPCMs sample (5 g) was put on a constant temperature heating plate with a temperature of 100 °C in 60 s. The spectrum and amount of thermal radiation depend strongly on an object’s surface temperature. This makes it possible for a thermal imaging camera to display the surface temperature of a microPCMs sample. The radiation depends not only on the temperature of the environment, but is also a function of the emissivity of the microPCMs. The emissivity (e) was set to 0.9. The thermal conductivity values of microPCMs samples with various graphene contents were discussed by comparing the infrared thermographs.

### 2.8. Differential Scanning Calorimeter (DSC) Tests

The thermal analyses of microPCMs were measured by a Perkin-Elmer Diamond DSC (USA) at a heating rate of 5 °C·min^−1^ between the range of −20 to 50 °C under nitrogen (*N*_2_) atmosphere. The encapsulation efficiency of the core material content was measured from the heat of fusion (Δ*H*_f_) of PCM [22]. The method to evaluate the encapsulated ratio (*E_r_*) is by Equation (2),

(2) $$E_{r}=\frac{\Delta H_{f,microPCMs}}{\Delta H_{f,PCM}\times m_{PCM}}\times 100\%$$

where $\Delta H_{f,microPCMs}$ is the calculated heat fusion of microPCMs by DSC, $\Delta H_{f,PCM}$ the latent heat of fusion of PCM, $m_{PCM}$ is the total weight of PCM in emulsion. The $\Delta H_{f,microPCMs}$ value of the microPCMs, was calculated through the integral of the curve area by software.

## 3. Results and Discussion

### 3.1. Self-Assembled Mechanism and Morphologies of microPCMs

**Figure 1:**
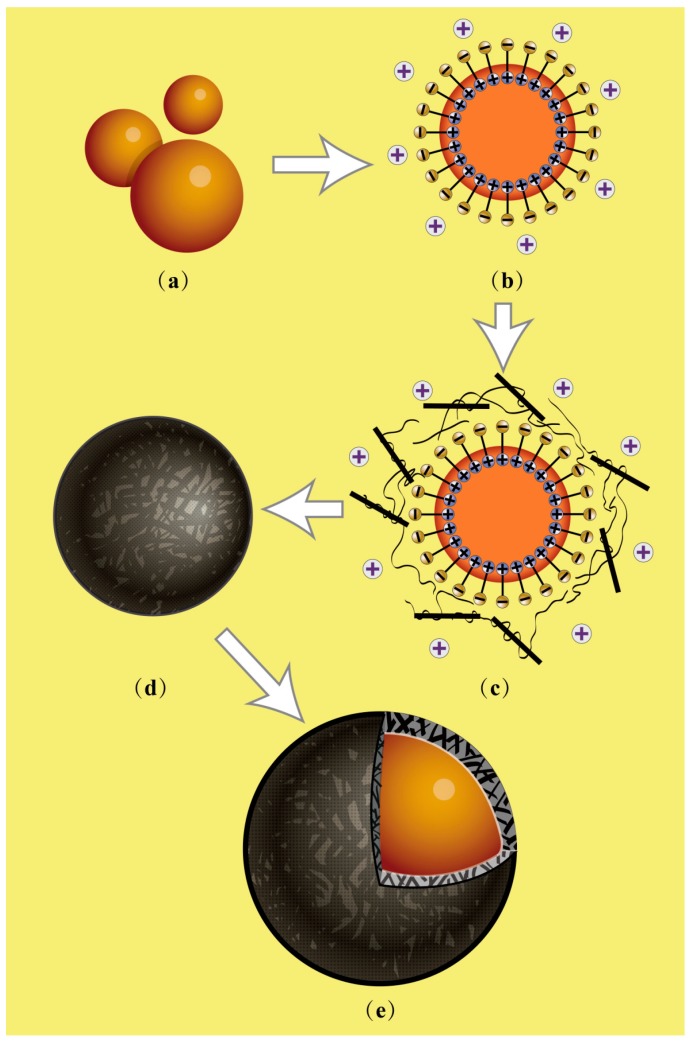
Illustration of microcapsules containing phase change materials (microPCMs) fabricated by a self-assembled process, (**a**,**b**) paraffin was emulsified by styrene maleic anhydride (SMA) molecules; (**c**) the mixture of melamine-formaldehyde (MMF) prepolymer and graphene added dropwise, MMF prepolymer and graphene absorbed on core droplets by electric charge; (**d**) microcapsules formed through a polymerization process; and (**e**) the state of graphene in the shell.

In this work, microPCMs samples were fabricated using MMF prepolymer as the shell material through an in-situ polymerization. Normally, melamine-formaldehyde (MF) is widely applied to prepare various functional microcapsule products due to its low price, easy technology, high compactness, fire resistance, and chemical resistance [22]. However, the residual formaldehyde in crosslinked MF resin is harmful to health and the environment. By contrast, there is nearly no residual formaldehyde remaining in the crosslinked MMF resin because of sufficient group reaction [21]. MMF prepolymer also has been proved to be suitable for preparing microcapsule products [23]. SMA was applied as a disperse agent to emulsify the oily paraffin into core droplets. Figure 1 illustrates the fabrication process of microPCMs in emulsion state. SMA molecules can be hydrolyzed when the pH value of the emulsion is less than pH 7. SMA molecules are charged negatively, and the oil phase material is dispersed in a certain aqueous solution forming an O/W emulsion under mechanical stirring (Figure 1a). Then SMA hydrolyzed molecules are subsequently attached to the interface of the inter phases, and the lipophilic ends extend into the interior of the droplets and the hydrophilic ends of the carbonyl groups extend to the aqueous phase [21,22,23]. Core droplets are ultimately separated through the regulation of hydrolyzed SMA molecules (Figure 1b). Thus the surfaces of the oil droplets are negatively charged. The prepolymer MMF has functional groups of amine, imine, and –N–CH_2_OH, which dissolve with negative charge in an acid aqueous solution combining with hydrogen ions. Therefore, prepolymer can be absorbed onto the droplet surfaces by electrostatic interaction (Figure 1c). Interestingly, it has been proved that inorganic nanoparticles can be absorbed onto the droplets by promoting the effects of chain entanglement and electrostatic attraction [16]. Similarly, graphene is absorbed onto the surface of the droplets by both forces. At an equilibrium point, MMF-prepolymer molecules are crosslinked to form the nano- inorganic/organic shells under a high temperature and correct pH value (Figure 1d). The shells have a self-assembled hybrid structure with a solid defined strength (Figure 1e).

**Figure 2:**
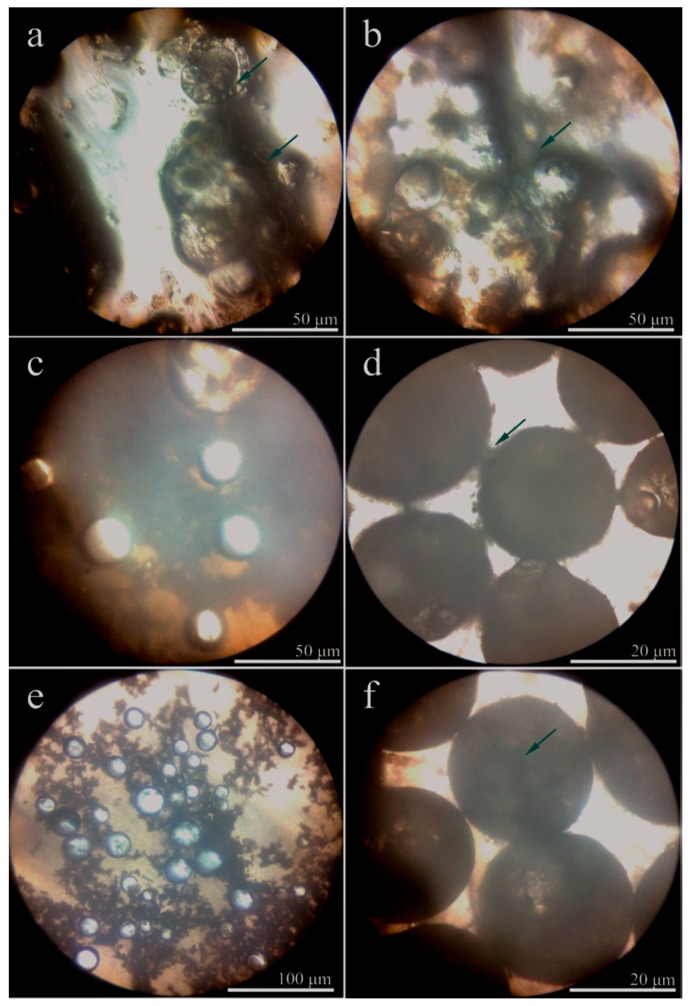
Optical microscope morphologies of microPCMs in emulsion, (**a**,**b**) core droplets emulsified by SMA molecules; (**c**–**e**) the shell forming process; and (**f**) the final states of microPCMs in the emulsion.

The above self-assembled mechanism of graphene hybrid shells already has been proved in previous work [23]. In order to further understand the self-assembled process of microPCMs, the morphologies of emulsion in various states were directly observed by an optical microscope (Figure 2). The Figures possess various scale bars to show details more clearly in the different fabrication steps. The oily paraffin is dispersed into core droplets by SMA with a stable state in the emulsion (Figure 2a,b). Lipophilic ends and hydrophilic ends help to keep the SMA hydrolyzed molecules attached to the interface of droplets. With the addition of prepolymer MMF, the molecules can be absorbed onto the surface of the droplets through electrostatic interaction (Figure 2c). In addition, graphene is absorbed onto the droplets surface with the help of both chain entanglement and electrostatic attraction [14]. The color of the microPCMs is black (Figure 2d) indicating that the graphene forms a hybrid with the polymer forming the inorganic/organic composite shells. The substances in the emulsion decrease with the development of polymerization. In other words, this means that prepolymer and graphene in the emulsion have been consumed to form hybrid shells (Figure 2e). Finally, the end microPCMs product is achieved by washing to remove impurities. It can be seen that these microcapsules have a regular globe shape with a smooth surface without a break (Figure 2e). They have a dark color with an average diameter of 20 μm. Almost no adhesion or impurity substances exist between the different microPCMs particles.

**Figure 3:**
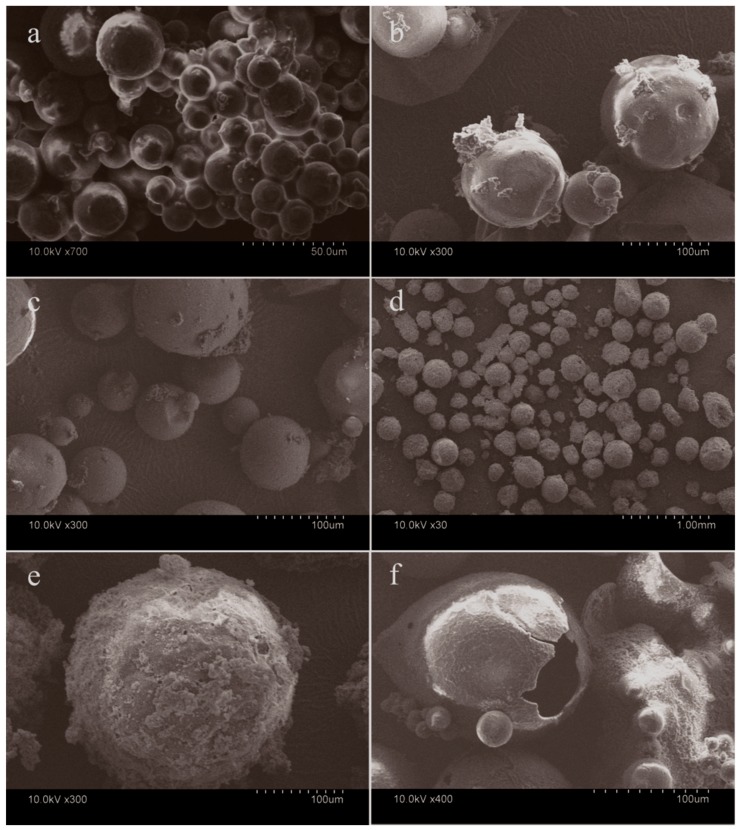
Scanning electron microscopy (SEM) morphologies of microcapsules with various graphene contents, (**a**) MG-0; (**b**) MG-2; (**c**) MG-4; (**d**) MG-6; (**e**) MG-8, and (**f**) a typical microcapsule with a broken shell.

Usually, microcapsule products have four basic characters which are surface morphology, mean size, shell thickness, and shell strength [11]. For the self-assembled polymerization method, these characters normally can be controlled by regulating the polymerization parameters such as the emulsion-stirring rate, prepolymer adding rate, and core/shell weight ratio [12]. For example, the morphology of microcapsules is smoother with a lower addition rate of shell material [5]. Also, the shell strength can be controlled by the prepolymer adding rate and core/shell weight ratio [24]. In previous works, the mechanical properties of single microcapsules were systemically investigated using a nanoindentation method [24]. It was found that higher strength and high compactness for shells can be achieved with a lower dropping rate of shell materials. The reason is that the lower dropping rate can result in the shells forming slowly and thus avoiding defects on the shells as much as possible. The emulsion stirring rate determines the mean size of the microcapsules. The mean size decreases with increasing stirring rate of the emulsion [14]. With the same mean size for the microcapsules, their thickness of shells is definitely determined by the core/shell ratios [7]. More related results can be found in previous works on the design of the structure of microcapsules [25]. To simpify this character parameter of microcapsules, the stirring rate in this work was set as a fixed value of 3000 r·min^−1^. Five typical microPCMs samples were prepared with different graphene/MMF percentage of 0%, 2.0%, 4.0%, 6.0%, and 8.0%, which are coded as MG-0, MG-2, MG-4, MG-6, and MG-8, respectively. As all microPCMs samples were fabricated under the same emulsion rate, it was considered that they had the same mean size. For the inorganic/organic hybrid microcapsules, the additive of the nanoparticles can greatly influence the morphologies of the shells. For example, Su [16] proved that the surface morphology of microcapsules was greatly affected by the contents of CaCO_3_ nanoparticles adhering on the shells. In this work, more morphology details of the shells can be observed from the SEM morphologies of the microcapsule samples (MG-0, MG-2, MG-4, MG-6, and MG-8) with various graphene contents. Microcapsules (GM-0) have a global shape without a break (Figure 3a), which is consistent with the previous results [14]. The microcapsule samples (GM-2, GM-4, GM-6) have a very smooth surface without attachment (Figure 3b,c). The shell thickness and the shell structure can be recognized from the break shells (Figure 3d). On the contrary, the microcapsules (GM-8) have a rough surface and impurity attachment adheres onto the shells (Figure 3e). It indicates that not all the graphene can be consumed during the shell formation process. Microcapsules (GM-6) may have the largest graphene deposition amount on the shells. Because the polymer molecules tangle with graphene, the unconsumed polymers will lead to some graphene remaining in the emulsion. The core-shell structure of the microcapsules can be easily seen from a typical microcapsule with a broken shell (Figure 3f). The shell has a thin membrane structure protecting the core material. The thickness of the shell has a uniformity value, which can be evaluated through the scale. In order to measure the shell thickness, microcapsules were usually mixed in gelatin gel to form a composite sample [25]. The dried composite was cut to obtain the cross-section with an ultramicrotome. Because the microcapsules cannot all be cut through the equator of the globe microcapsules, the shell thickness was an average value of 50-times measurement results for each microcapsule sample.

**Figure 4:**
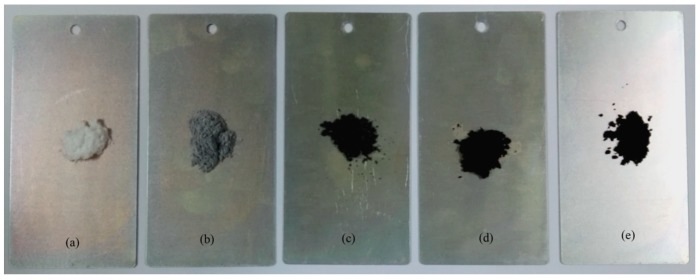
Photographs of microPCMs samples, (**a**) MG-0; (**b**) MG-2; (**c**) MG-4; (**d**) MG-6, and (**e**) MG-8.

Graphene has a natural black color. The colors of microcapsule samples (GM-0, GM-2, GM-4, GM-6, and GM-8) are shown in Figure 4. GM-0 sample has a white color (Figure 4a). The black color of microcapsule samples becomes deeper and deeper (GM-2, GM-4, GM-6, GM-8, and GM-10) (Figure 4b–e). This indicates that more graphene has successfully deposited on increasing the graphene/polymer ratios in the shells base on the color changes. The hybridization process and electric charge may determine the graphene deposition on the shells. Table 1 lists the physical structure characters of microcapsules fabricated under an emulsion stirring rate of 3000 r·min^−1^. With the same stirring rate, the microcapsule samples nearly all have the same mean size values in a range of 25.0–27.8 μm. Moreover, the microcapsules have shell thickness values between 3.1 μm and 3.5 μm without large change. It has been reported that the shell thickness is mainly determined by the core/shell ratio [25]. The microcapsule samples have the same core/shell ratio of 1/1 in this work. As microcapsule formation in this study is a coacervation process, the addition of nano-particles may greatly affect the morphology of the shells [14,16]. With increasing graphene content, the data indicates that the addition of graphene does not affect the shell thickness. This result is similar to that of microcapsules with nano-CaCO_3_/polymer shells. It was found that the nano-CaCO_3_ particles were embedded into polymers to form a stable composite shell structure. Because of the same fabrication mechanism, the microPCMs may have the same polymerization process as the previous products. It can be imaged that graphene adheres to the core droplets with MMF to form inorganic/organic composite shells.

### 3.2. Chemical Structure of microPCMs

**Figure 5:**
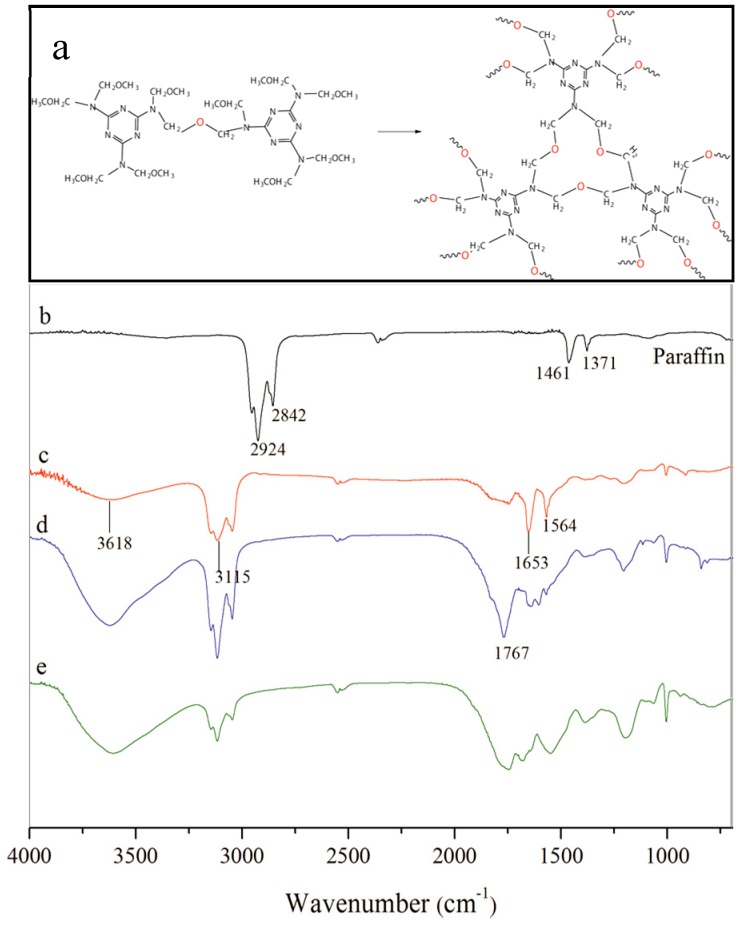
Chemical structural of microPCMs samples, (**a**) chemical formula of MMF prepolymer crosslinking process; FT-IR curves of microPCMs samples: (**b**) pure paraffin; (**c**) MG-2; (**d**) MG-4, and (**e**) MG-6.

MMF prepolymer has a crosslinked chemical reaction in the emulsion state to yield a compactable shell structure. The prepolymer interaction generated oligomer is shown in Figure 5a. Under acid conditions, the MMF prepolymer undergoes a dehydration reaction through methyl oxygen (–OCH_3_) groups. The further cross-linking reactions of the oligomer form the stable shells of the microcapsules. The crosslinked MMF network provides a protective barrier between the core material and the external environment preventing core material leakage or contamination. The marked FT-IR spectra belong to (*b*) paraffin, (*c*–*e*) microPCMs samples (MG-2, MG-4, and MG-6). The spectrum of paraffin (*b*) exhibits the characteristic absorption bands at around 1461 cm^−1^ and 2924 cm^−1^ attributed to –CH_3_ asymmetric bending vibration and alkyl –CH_2_ stretching vibration. The spectra of all microcapsule samples (*c*, *d,* and *e*) exhibit three absorption bands at around 1653 (C–C), 3115 (N–H) and 3618 cm^−1^ (O–H). The characteristic peaks of the paraffin no longer exist, which indicates that the paraffin has been totally microencapsulated. The spectra also imply that microcapsule samples have different graphene contents in the shells. Based on the intensity of absorption peaks, it was found that both samples have O–H bonds, which may belong to the residual water. More graphene in the shells does not change the chemical structure of the graphene/MMF composites. In other words, the graphene has a physical bond with crosslinked MMF networks.

### 3.3. Determination of Graphene in the Shells

**Figure 6:**
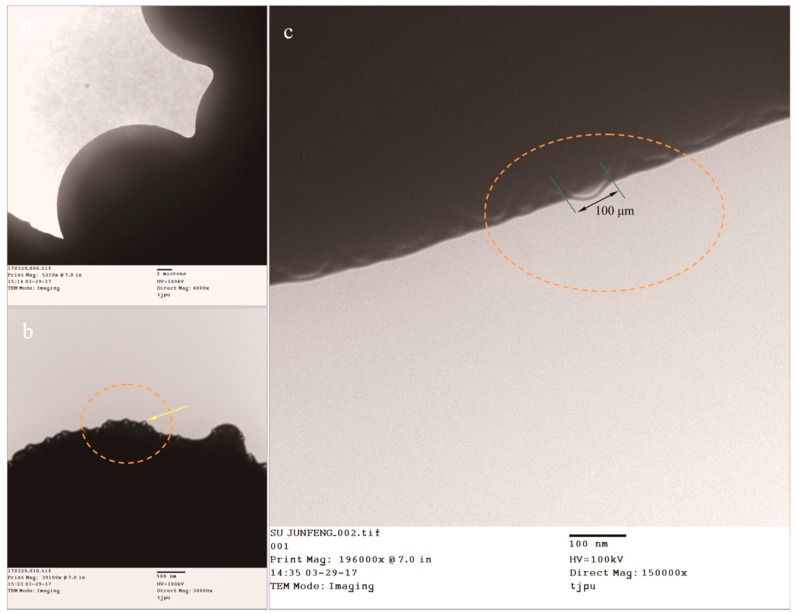
Transmission electron microscopy (TEM) morphologies of the microPCMs sample (MG-2), (**a**) the microPCMs particles on a copper screen; (**b**) a hybrid shell surface with graphene; and (**c**) typical graphene extending out of the shell surface (100 nm).

In previous work, it was confirmed that graphene can exist in the shells of microcapsules [14]. However, more details of the microstructure of graphene/MMF hybrid shells need to be determined by analysis, because understanding of the microstructure will be helpful in knowing the graphene states affecting the thermal properties. The microcapsules states can be directly observed on a copper screen through the TEM morphologies (Figure 6a). The surface morphology of a single microcapsule is shown in Figure 6b after magnification, in which the arrows point to some particles extending out of the shell. These particles show the existence of graphene, the impurity substances have been removed before tests. This conclusion can be further confirmed by an enlarged morphology (Figure 6c). Interestingly, a sheet structure is found with a size of 100 nm, its geometrical characteristics are consistent with monolayer graphene.

**Figure 7:**
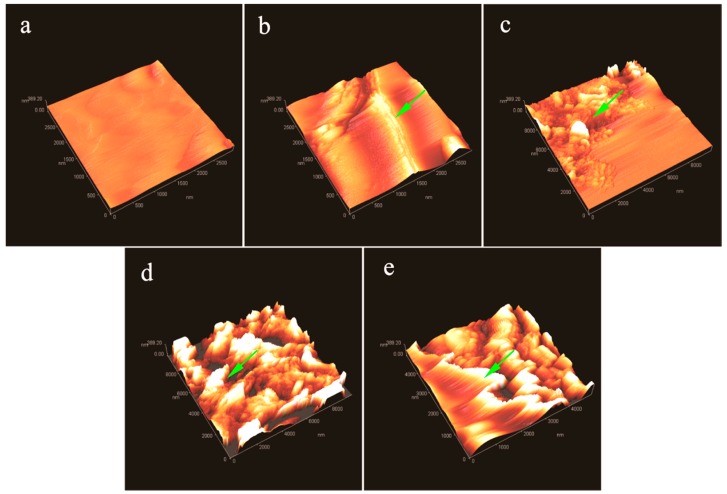
Atomic force microscopy (AFM) morphologies of microPCMs shells with/without graphene, (**a**) MG-0; (**b**) MG-2; (**c**) MG-4; (**d**) MG-6, and (**e**) MG-8.

More information about the graphene/MMF hybrid microstructure of shells can be found in the AFM morphologies of a single microcapsule surface with/without graphene. GM-0 sample has a relatively rough shell (Figure 7a). For GM-2, GM-4, and GM-6 samples, graphene can be identified by their AFM morphologies (Figure 7b–d). These shells have more smooth surfaces conforming to the previous SEM results. A lamellar structure can be found with a size less than 200 nm, which is attributed to the graphene. Moreover, more lamellar structures appear in shells on increasing the graphene amount (GM-2, GM-4, and GM-6). This indicates that more graphene has been deposited on the shells with more graphene addition in the shell formation process, which is consistent with previous SEM and TEM results. Graphene is difficult to disperse in an absolute uniform state in an emulsion by ultrasonic dispersion [14]. Therefore, some graphene layers are easily piled together by electrostatic attraction. Furthermore, a graphene/MMF hybrid structure can be identified from an enlarged AFM morphology of GM-6 (Figure 7d) as the arrows point to. The shells have an organic/inorganic composite structure, which may be helpful to enhance the properties of microcapsules.

**Figure 8:**
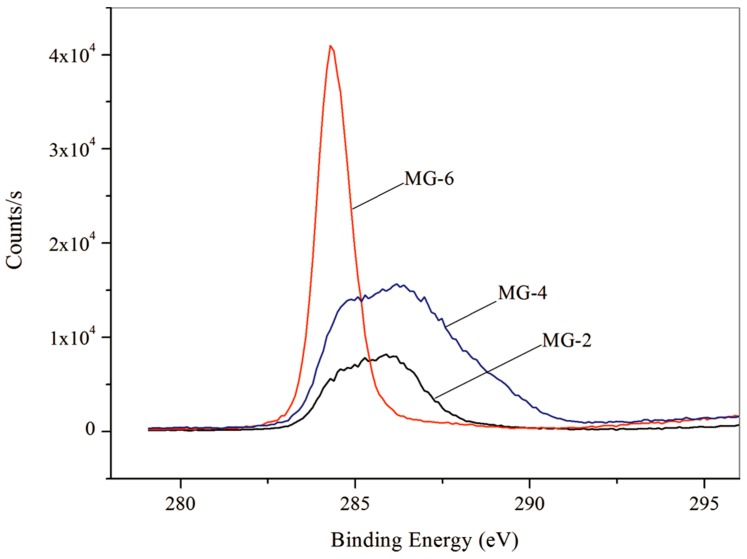
X-ray photoelectron spectroscopy (XPS) curves of microPCMs samples (MG-2, MG-4, and MG-6).

In order to further verify the contents of graphene in shells, XPS spectra were analyzed to give more structure details on the carbon element in the microcapsule shells. Figure 8 shows the XPS spectra (C1s) of GM-2. Three functional groups can be found, they are the non-oxygenated C–C (285.08 eV), the ether C–O (286.43 eV), the C=N bond (289.00 eV). Another C state is found at 284.40 eV belonging to the graphene. A similar results has been reported on the binding energy at 284.40 eV assigned to graphene [26]. The XPS results prove that the graphene has deposited on the microcapsules surface. In our future work, the charge adsorption and chain entanglement phenomena will be investigated systematically, which will balance the amount of graphene deposition on the shells.

### 3.4. Thermal Stability of MicroPCMs

**Figure 9:**
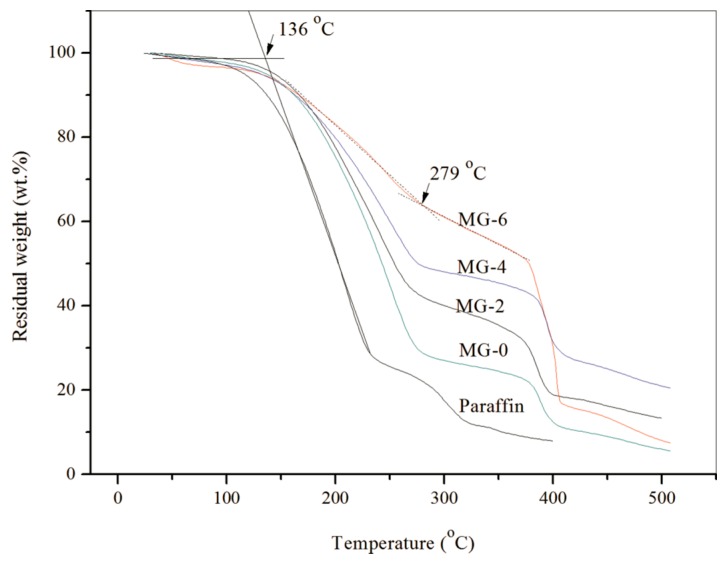
Thermogravimetric analysis (TGA) curves of pure paraffin and microPCM samples.

TGA curves were analyzed to study the thermal stability of pure paraffin and the microPCMs samples (GM-0, GM-2, GM-4, and GM-6) as shown in Figure 9. Pure paraffin loses its weight sharply from 136 °C. This result is similar to the previously reported character [10,11,12]. GM-0 loses weight in a temperature range of 200–400 °C. Its TGA curve has a two-step weight losing process. In the first step, the weight of the microcapsules decreases sharply from 200 °C, which is attributed to the weight loss of residual micro-molecular organics in the microcapsules. At the same time, the microcapsules are broken and release the oily small molecules of paraffin with increasing temperature. Then the paraffin decomposes rapidly. The second step happens in a temperature range between 393 °C and 473 °C, which is caused by the decomposition of the microcapsules shells. Comparing to GM-0, the beginning weight loss rate of GM-2 has obvious increased, which implies that its thermal stability has been improved. Before the temperature of 385 °C, the weight loss curve has a small slope. It means that the shells still have a compactable structure without rapture. The weight loss is about 15% before 200 °C and can be attributed to the residual water or small molecules in the shells. The TGA curve has a peak at 399 °C, which can be considered as the moment when most of the microcapsules have broken and released the paraffin. At such a high temperature, the paraffin molecules have decomposed rapidly. The reason for the above phenomena can be attributed to two facts. First, the graphene possesses an excellent heat transfer performance, which leads to a uniform heat distribution in the microcapsules and at the same time enhances the thermal stability of the microcapsules. The weight loss curve is nearly horizontal at about 500 °C, which points to the fact that all organic materials have been decomposed. Second, the graphene/MMF composite structure can enhance the thermal stability of the microcapsules. A similar result has already been reported in previous works on other microcapsules with nano-particles/polymer shells [16]. The TGA curves of GM-4 and GM-6 have a similar shape with GM-2. This phenomenon means that most of the microcapsules have been broken and released the paraffin at an even higher temperature. More graphene can enhance the thermal stability of the microcapsules. The GM-6 sample has a residual testing weight of about 17.41% at 500 °C. The final residual weight belongs to the total weight of organic carbonization and graphene.

**Figure 10:**
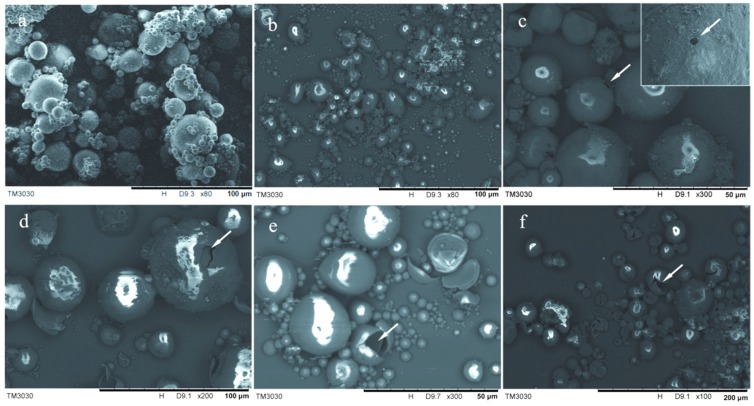
SEM morphologies of microPCMs sample (MG-2) at various temperatures, (**a**) 100 °C; (**b**) 150 °C; (**c**) 200 °C; (**d**) 250 °C; (**e**) 300 °C and (**f**) 350 °C.

In order to understand more details of the thermal states of the microcapsules, the surface morphologies of the microcapsules were observed after suffering a high temperature process for 10 min. Figure 10a–f shows the SEM morphologies of MG-2 sample at various temperatures of 100 °C, 150 °C, 200 °C, 250 °C, 300 °C, and 350 °C. As shown in Figure 10a,b, nearly all the microcapsules have survived at a temperature of 150 °C. However, a few of microcapsules have holes or cracks on the shells by the heat at a temperature of 200–300 °C (Figure 10c,d). Comparatively, microcapsules cannot resist higher temperature at 300–250 °C as shown in Figure 10e. Some empty microcapsules remained with only shells after volatilization.

**Figure 11:**
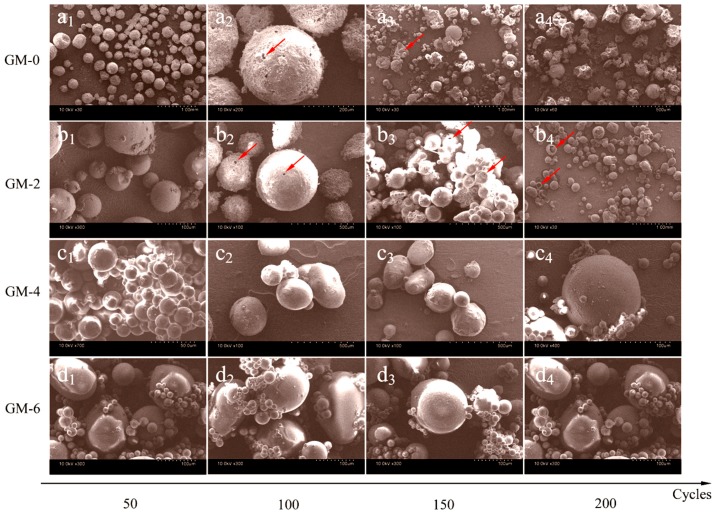
SEM morphologies of microPCMs samples (MG-0, MG-2, MG-4, and MG-6) with various heat absorbing-releasing cycles (50, 100, 150, and 200 cycles): (**a**_1_–**a**_4_) MG-0; (**b**_1_–**b**_4_) MG-2; (**c**_1_–**c**_4_) MG-4; and (**d**_1_–**d**_4_) MG-6.

Another method was designed to test the thermal stability of microPCMs under heat absorbing-releasing cycles using a temperature-controlled chest. The samples were heated to 50 °C with a rate of 2 °C·min^−1^ and maintained for 10 min, and then the temperature decreased to −10 °C·min^−1^ with a rate of 2 °C·min^−1^. This thermal treatment cycle for each sample was repeated for 50, 100, 150, and 200 times. Figure 11(a_1_–a_4_) shows the SEM morphologies microPCMs (MG-0) after 50, 100, 150, and 200 cycles, respectively. Before 100 cycles, the microcapsules still kept a compact structure. However, a lot of the microcapsules were broken after 200 cycles without resisting the heat treatment. Comparatively, MG-2 particles survived after 200 cycles Figure 11(b_1_–b_4_). Only a few microcapsules had holes on the shells. This conclusion implies that the microcapsules with graphene have good thermal stability when suffering a violent temperature change. Figure 11(c_1_–c_4_) and Figure 11(d_1_–d_4_) show the SEM morphologies of microPCMs (GM-4 and GM-6) after 50, 100, 150, and 200 cycles, respectively. All microcapsules from beginning to end, maintain the global shape without breaking. Graphene in the shell can dramatically help to resist the temperature changes avoiding the rupture of the shells.

### 3.5. Thermal Conductivity of microPCMs

**Figure 12:**
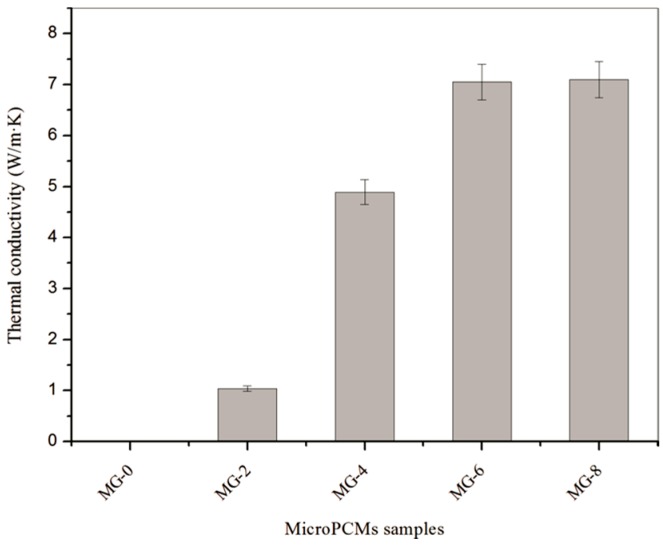
Thermal conductivity values of microPCMs samples.

In this study, the microPCM samples with graphene were expected to retain remarkable thermal conductivity, which gives the microcapsules a faster heat absorbing-releasing capability during thermal exchange. The thermal conductivity values of five samples (GM-0, GM-2, GM-4, GM-6, and GM-8) were measured to investigate the influence of the graphene amounts (Figure 12). First, it is found that GM-0 possesses a very low thermal conductivity of 0.2 W/(m·K). As a polymer material, the organic molecules greatly resist heat transfer through the core-shell structure. Secondly, the thermal conductivity values (GM-2, GM-4, GM-6. and GM-8) increase with increasing graphene content in the shells. For example, the thermal conductivities of GM-2 and GM-4 are about 1.05 W/(m·K) and 4.95 W/(m·K). On the other hand, GM-6 and GM-8 nearly have the same thermal conductivity of 7.08 W/(m·K). These data indicate that graphene can significantly enhance the thermal conductivity of microPCMs because of the excellent thermal conductivity of graphene in microcapsules. The mixed inorganic particles can enhance the thermal conductivity. In the inorganic/organic composites, the thermal transition barrier is reduced by the graphene partly forming heat transition bridges. On the other hand, the thermal conductivity of the microcapsules cannot increase any more to a certain degree with adding more graphene. In future works, the perfect amount of graphene will be modeled to optimize the microstructure of the graphene/organic hybrid shells for heat conductivity.

IRT has been widely applied to measure the radiant thermal energy distribution and heat conduction behavior emitting from a target. For example, this technology is employed to complete the non-destructive evaluation or non-destructive devaluation applied to composite structures [27]. Furthermore, IRT is particularly useful to detect the material structure at micro-scale level. Such examples have been carried out to examine the thermal patterns in micro-arrays, micro-tubes, and micro-channel heat sinks. It has also been reported that the same tool can be used to characterize the physicochemical and mechanical properties of microPCMs [28]. Particularly, it was used to understand the influence of axial and radial direction temperature distributions. The IR camera can directly capture photos or videos in a sequence, which reveals information about the heat distribution on a material surface in a fixed area at different temperatures. The IR images with different colors correspond to the temperature distribution of the material sample surface. The thermal conductivity characters can be comparatively analyzed by verifying the colors at different times [29].

**Figure 13:**
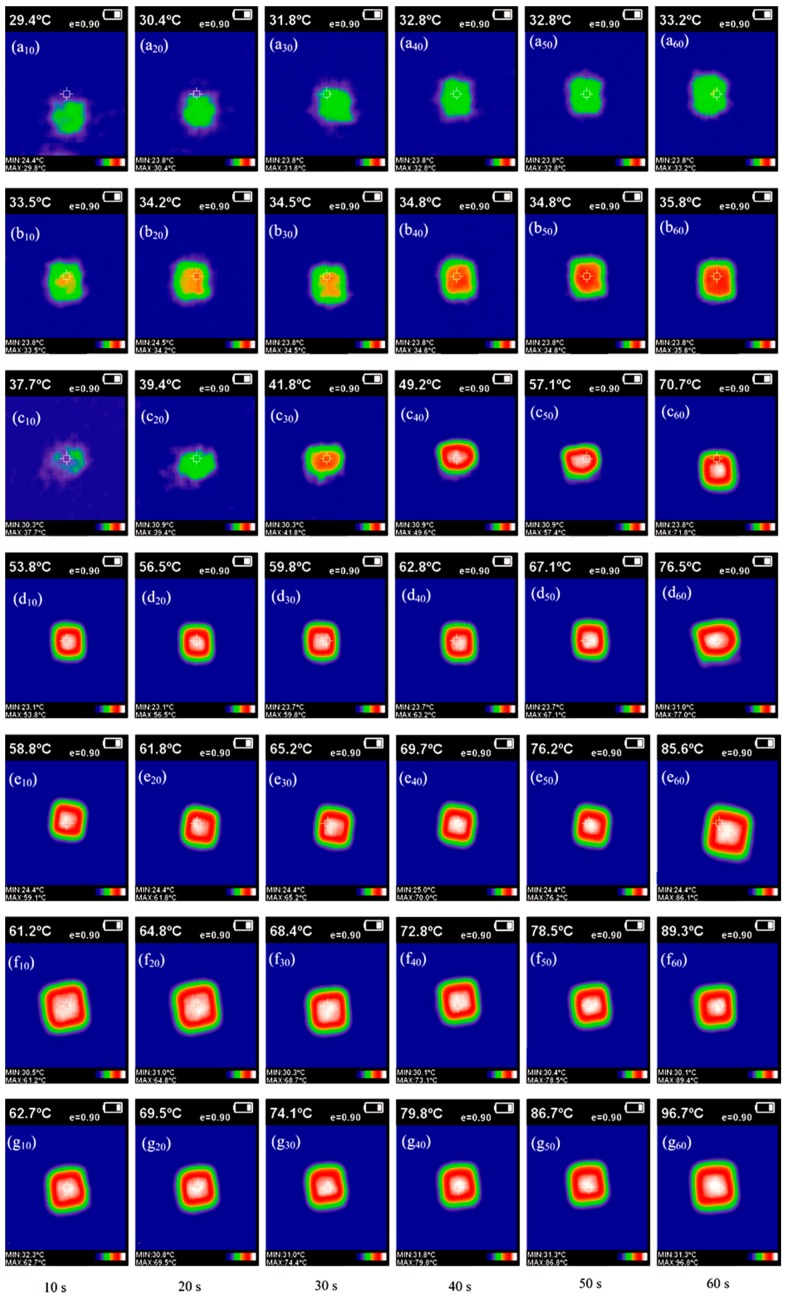
Infrared thermograph (IRT) results of the thermal transmission of various microPCM samples with/without graphene, each microPCM sample (5 g) was put on a constant temperature heating plate with temperature of 100 °C in 60 s, (**a_10_**–**a_60_**) MG-0; (**b_10_**–**b_60_**) MG-1; (**c_10_**–**c_60_**) MG-2; (**d_10_**–**b_60_**) MG-4; (**e_10_**–**e_60_**) MG-6; (**f_10_**–**f_60_**) MG-8; and (**g_10_**–**g_60_**) MG-10.

Figure 13 shows the IR images of seven microPCMs samples with/without graphene over 60 s on a constant temperature heating plate (100 °C). MG-0, MG-1, MG-2, MG-4, MG-6, MG-8, and MG-10 are labeled as a, b, c, d, e, f, and g, respectively. MG-1 (1.0% graphene in shell) and MG-10 (1.0% graphene in shell), not listed in Table 1, were also fabricated to increase the continuity of the samples. It is helpful to understand the effect of graphene content on the thermal conductivity of the various samples. Six images for each microcapsule sample were captured at intervals of 10 s, which are labeled as 10, 20, 30, 40, 50, and 60 in subscripts. All IR images were obtained at the same emissivity (*e*) value of 0.9, which is noted on top of each image. The reason is that the MMF polymer usually possesses an *e* value between 0.90 and 0.98 [30]. Because IRT is only a technology to inspect solid structures and is also considered a ‘boundary technique’, this means its ability for inspection is limited to a certain depth of the target to subsurface defect detection [27]. Therefore, the microPCM particles were piled together with the same area and height avoiding the influence of the sample’s shape. It was detected first that the MG-0 sample without graphene had a poor thermal conductivity. In the 60 s process of raising the temperature, MG-0 changed its temperature from 25.0 °C (room temperature) to 33.2 °C with a calculated heating rate of 0.137 °C/s. The IRT color of the sample still stays green in 60 s without an obvious warming zone (Figure 13(a_10_–a_60_)). Comparatively, MG-0 has a color transformation from green to red in 60 s. Its temperature also has risen from 25.0 °C to 35.8 °C (Figure 13(b_10_–b_60_)). This phenomenon indicates that only 1.0 wt % graphene in the shell material can evidently enhance the thermal conductivity of the microPCMs particles. This trend also appeared in IR images of other microPCMs samples. The final temperatures at the end of 60 s of these samples are 70.7 °C (Figure 13(c_10_–c_60_)), 76.5 °C (Figure 13(d_10_–d_60_)), 85.6 (Figure 13(e_10_–e_60_)), 89.3 °C (Figure 13(f_10_–f_60_)) and 96.7 °C (Figure 13(g_10_–g_60_)), which is a sharp increase of temperature. It means that microPCM samples have a dramatic enhancement of thermal conductivity with increasing graphene contents in the shells. This conclusion also can be drawn from the temperature data at 10 s for these samples. In images (b_10_, c_10_, d_10_, e_10_, f_10_, and g_10_) after only a short time, the samples changed to 33.5 °C, 37.7 °C, 53.8 °C, 58.8 °C, 61.2 °C and 62.7 °C. These results are at the same time consistent with the above analysis of the data of direct measurements for thermal conductivity.

### 3.6. Thermal Storage Characteristics of MicroPCMs

**Figure 14:**
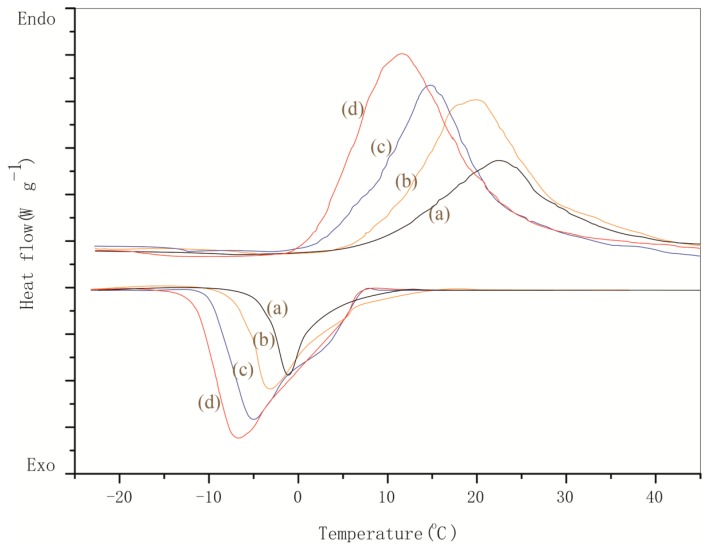
Differential Scanning Calorimetry (DSC) curves of microPCM samples with/without graphene, (**a**) MG-0; (**b**) MG-2; (**c**) MG-4 and (**d**) MG-6.

DSC tests were carried out to analyze the thermal energy storage capacity of the microPCM samples in terms of melting/solidifying temperatures and latent heat capacity. Figure 14 shows the DSC curves of microPCMs samples (MG-0, MG-2, MG-4 and MG-6) in the charging and discharging process. Paraffin used in this work has a phase change temperature at 19.2 °C [11]. In general, phase change materials have a phase transition temperature range. A phase transition temperature is usually obtained by tangent treatment of the DSC curve. One peak exists for each charging and discharging curve within a temperature range of −20 °C to 40 °C. These peaks are obviously caused by the melting and solidifying of paraffin. This conclusion also has been proved in other previous works about microPCMs [14]. For the MG-0 sample without graphene, its melting phase change temperature (*T*_m_) is about 20.2 °C similar to the *T*_m_ of pure paraffin. It means that the polymeric material shells do not greatly influence the phase change behavior of paraffin. With the addition of graphene in the shells, microPCM samples of MG-2, MG-4, and MG-6 have *T*_m_ values of 18.4 °C, 16.2 °C and 10.5 °C, respectively. These temperatures are even lower than the *T*_m_ value of pure paraffin. With the increasing of graphene content in the shells, the *T*_m_ values of the microPCM samples (MG-2, MG-4, and MG-6) decreased sharply. These phenomena imply that the graphene has dramatically enhanced the thermal sensitivity of the microencapsulated paraffin. Heat will rapidly transfer through the graphene composite shells with pure paraffin inside. Because the microcapsules have a large specific surface area, paraffin can absorb more thermal energy with less thermal resistance. Graphene can greatly enhance the smoothness of heat transfer at the same time through a heat channel formed by overlapping graphene. The slopes of the DSC curves increased with the more graphene added in the shells during the melting process of the phase change. Meanwhile, the intervals of the phase transition temperatures became narrower. All these results indicate that the addition of graphene can significantly enhance the heat transfer efficiency. In addition, the solidification temperatures of MG-2, MG-4, and MG-6 (−3.5 °C, −6.1 °C, −7.9 °C) decreased compared to MG-2 (−1.2 °C) without graphene. This proves in a further way that the graphene can improve the thermal sensitivity of microPCM samples.

Based on the above DSC curves, the melting energy (Δ*H*_m_) and solidifying energy (Δ*H*_s_) are listed in Table 2 calculated through area integrals of the peaks. MG-0, MG-2, MG-4, and MG-6 have Δ*H*_m_ values of 132.5, 145.2, 155.7, 160.5 J/g, and their Δ*H*_s_ values are 120.5, 138.5, 149.8, 158.7 J/g, respectively. With the graphene content increasing in the shells, the microPCM samples have an obvious enhancement of latent thermal energy storage capability. This conclusion is consistent with the analysis of the above DSC curves. The ratio relationship expression (*δ*) of melting/solidifying latent heat can be written as Equation (3),

(3) $$\delta =\frac{\Delta H_{m}}{\Delta H_{s}}$$

where *δ* is defined as the heat energy storage capability. The *δ* values represent a trend approximately equal to 1 for MG-0 (90.9%), MG-2 (95.4%), MG-4 (96.2%), and MG-6 (98.9%), which indicates further proof from another side that graphene has the capability of enhancing the thermal storage efficiency of microPCMs. Because the microPCMs nearly have similar *E_r_* values as shown in Table 1 calculated based on the DSC curves, the thermal storage efficiency can definitely exclude the influence factor of verifying the mass of paraffin. In the future, this excellent thermal storage capability gives these graphene microPCMs wider application prospects for practical applications such as intelligent wear products, solar energy, buildings, and heat recovery systems.

## 4. Conclusions

The microPCM particles containing paraffin were successfully prepared by a self-assembled synthesis method with graphene/MMF hybrid shells. The microstructure and thermal reliability of various microPCM samples were investigated to understand the effects of graphene on the properties. The following conclusions can be drawn,

(1) The size, shell thickness, morphology, and chemical structure of microPCMs were investigated. MMF had a crosslinkage reaction forming a hybrid structure with graphene. It was found that the microPCMs were spherical particles and the graphene enhanced the smoothness degree of the shell surface.
(2) The SEM, TEM, AFM, and XPS results ensured the existence of graphene in the shells. It was found that graphene hybrid shells were constructed though forces of electric charge absorption and long-molecular entanglement.
(3) TGA results indicated that the graphene additive greatly enhanced the thermal stability of microPCMs. The heating-cooling tests also proved that the microPCMs with more graphene in the shells resisted violent temperature changes without damage.
(4) The thermal conductivity tests indicated that the phase change temperature of microPCMs was regulated by the graphene additive because of the reduction of the thermal transfer hindrance of the hybrid shells. At the same time, IRT results also implied that the microPCMs with more graphene had a higher heat transfer capability.
(5) DSC tests proved that the latent thermal energy capability of microPCMs had been improved with a higher heat conduction rate. In addition, infrared thermograph observation implied that the microPCMs had a sensitivity response to heat during a phase change cycling possess because of the excellent thermal conductivity of graphene.

## Figures and Tables

**Table 1:** Characters of microcapsules with various graphene contents in the shells.

Microcapsules Sample	Core/Shell Weight Ratio	Graphene/Shell (wt %)	Emulsion Rate (r·min^−1^)	Mean Size (μm)	Shell Thickness (μm)
MG-0	2/1	0	3000	25.0	3.1
MG-2	2/1	2	3000	25.2	3.4
MG-4	2/1	4	3000	25.5	3.3
MG-6	2/1	6	3000	27.4	3.5
MG-8	2/1	8	3000	27.8	3.5

**Table 2:** The phase change data of microPCMs samples calculated through differential scanning calorimetry (DSC) curves.

microPCMs Samples	Melting		Crystallization		Heat Energy Storage Capability (%)
	*T*_m_ (°C)	Δ*H*_m_ (J/g)	*T*_c_ (°C)	90.9	
MG-0	20.2	132.5	−1.2	95.4	90.9
MG-2	18.4	145.2	−3.5	96.2	95.4
MG-4	16.2	155.7	−6.1	96.4	96.2
MG-6	10.5	160.5	−7.9	96.4	98.9
